# How to Provide Motherside Neonatal Resuscitation with Intact Placental Circulation?

**DOI:** 10.3390/children8040291

**Published:** 2021-04-08

**Authors:** David Hutchon, Simone Pratesi, Anup Katheria

**Affiliations:** 1 Emeritus Consultant Obstetrician, Memorial Hospital, Darlington DL3 6HX, UK; djrhutchon@hotmail.co.uk; 2 Neonatology Unit, Careggi University Hospital, 50134 Florence, Italy; 3 Neonatal Research Institute Sharp Mary Birch Hospital for Women & Newborns, San Diego, CA 92123, USA; Anup.Katheria@sharp.com

Immediate clamping and cutting of the umbilical cord have been associated with death and/or neurodisability [1,2,3,4,5]. Given the harm from immediate cord clamping it would seem logical that all infants should receive delayed cord clamping, but evidence for delayed cord clamping when resuscitation is required is limited. One approach would be to perform resuscitation while the cord is still intact. Several studies have demonstrated improvements in physiological outcomes, such as higher Apgar scores or oxygen levels in the first few minutes of birth with resuscitation with an intact cord compared to early cord clamping [6,7,8,9,10,11]. Another challenge, however, is implementation of this practice. While some groups have reported this practice to be relatively straightforward [11,12,13,14,15,16], others have struggled with its implementation [17]. Resuscitation equipment and procedures need to be acceptable to clinicians and parents for use outside a research environment [17,18,19]. Modifications of the equipment and increased co-operation between obstetric and neonatal teams are required. Practice and training are prerequisites to ensuring that resuscitation at the side of the mother with an intact placental circulation provides the same standard of care for the neonate and permit the obstetrical team to provide unhindered care for the mother. The care must meet International Liaison Committee on Resuscitation (ILCOR) [20] and other neonatal and obstetric standards, and adapt as new evidence emerges. For example, the guidance for the application of mask positive pressure ventilation in the poorly breathing neonate [21] may need to be reviewed in the light of evidence showing that mask application may actually precipitate apnoea and bradycardia [22]. Many preterm and some apparently asphyxiated term neonates will only require time to stabilise transition with an intact placental circulation while remaining on the resuscitation equipment. International guidelines for neonatal resuscitation suggest to delay cord clamping at birth for at least 30 s in preterm newborns whenever feasible, but there are no guidelines for neonatal providers [20]. This review aims to provide guidance for how neonatal providers can support placental transfusion for all infants, particularly the extremely preterm infant.

There are several resuscitation trolleys that are available for research use. Two are commercially available in Europe (CONCORD and LifeStart Trolley) and others are under research protocols (INSPIRE and NOOMA). No matter which is used, all should have some of the basic features as shown in Table 1.

## 1. Equipment for Neonatal Resuscitation with Placental Circulation Intact

Neonatal resuscitation equipment needs to be designed to allow for routine resuscitation with an intact placental circulation. At the same time the obstetric team needs to have access to provide any necessary care to for the mother. A stable platform which can be brought right up to the mother close enough for an intact cord may require modification and integration with the various designs of birthing tables. The practice of delayed clamping with resuscitation remains mostly as part of research protocols. This may be because concerns remain about the need for immediate resuscitation, or situations where there may be an imminent delivery without adequate time to setup equipment or space for extra personnel. Currently, two specially designed neonatal resuscitation trollies are available for resuscitation with an intact cord. While resuscitation with an intact cord can be achieved with standard equipment some of the above previously stated challenges may continue [23,24].

## 2. Design Features Required to Meet All Criteria for Current Standard Resuscitation

The precise requirements will vary between the two main modes of birth, assisted vaginal birth and caesarean section. While neonates born naturally sometimes need resuscitation this is infrequent and the equipment and procedure can be more readily dealt with. The equipment at assisted births needs specific requirements. A major consideration at caesarean section is maintaining the integrity of the sterile field. This needs the neonatal resuscitation team to be part of the sterile field. Provided nothing passes from the neonatal team to the obstetric team after the neonate is delivered, some compromise to the equipment used by the neonatal team may be acceptable.

### 2.1. Platform

The surface of the platform should be flat to provide an optimal position for an open airway (“sniffing position”). It should be at a height which allows the neonatal team to provide the care necessary. The surface should be soft and firm. It must be stable so that the neonate can be placed on the surface without any risk of the platform moving or risk of the neonate falling off the platform. The platform should be of a sufficient size to accommodate also large term neonates and must be capable of getting as close as possible to the vaginal introitus or caesarean incision, as the cord must not be under tension for it to remain functional. In case of caesarean section, it can be covered with sterile drapes as is normal for the Mayo table.

### 2.2. Platform Position and Access by Neonatal Team

**Figure 1:**
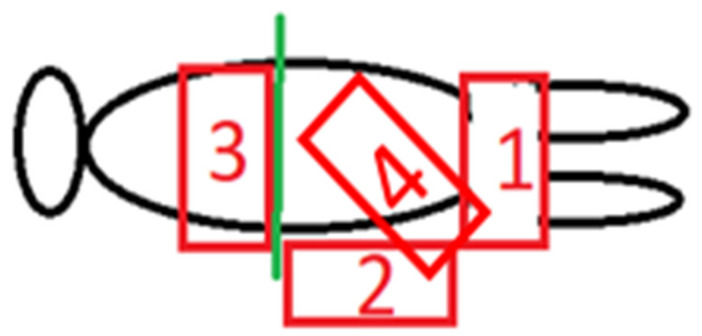
Possible positions for the platform during caesarean section.

With a specialized resuscitation trolley, three sides of the trolley can be easily accessed by a member of the resuscitation team. Figure 1 shows four possible positions for the platform during caesarean section.

*Position 1*, over the mother’s thighs seems the most straightforward position. Provided the platform is relatively thin, the surface can be virtually at the level of the uterus/placenta. It needs to be supported and not resting on the mother’s thighs. Only one person can have ready access to the neonate.

*Position 2*, at the side of the operating table is the normal position for the operating obstetrician, so this position requires the obstetrician to move away during the resuscitation. This position gives access for two or possibly three people around the resuscitation platform. The platform can be lowered to be significantly below the level of the placenta provided there is sufficient cord length. The surgical assistant remains to deal with the surgical wound and delivery of the placenta if necessary. Rarely will there be any significant problem during the few minutes after a birth. After about three minutes or earlier delivery of the placenta there is no physiological advantage for the resuscitation procedure to remain at the side of the mother.

*Position 3* is likely to place the neonate significantly above the placental level and may also require the obstetrician to move slightly if there is more than one person providing resuscitation. A sterile screen can be used to separate off the surgical field immediately after delivery of the baby. It is close to the face of the mother, who potentially could see, speak to and touch her baby.

*Position 4* is similar to position 1 but probably permit to manage newborn with a shorter cord. This position gives access for two people around the resuscitation platform but requires the obstetrician to move apart during the resuscitation.

In all four positions there needs to be an independent support for the platform which provides sufficient stability as well as mobility to get into position immediately after the birth and away again three to five minutes later. It needs to be able to accommodate height differences especially if it is also used at assisted vaginal birth. If the trolley is customised for caesarean birth only then the range of height adjustment is significantly reduced. The height of the operating table could be adjusted to meet the requirements of the resuscitation platform.

The resuscitation platform has significantly different height requirements for caesarean section as for assisted vaginal birth. Enterprising design is required to make the specialized equipment provide the same standards as routine resuscitation beds. At assisted vaginal birth it is inevitable that the platform to place the baby will be lower than the height of the standard resuscitation equipment. Raising the birthing table may help to reduce this compromise. The mother’s legs, in the lithotomy poles may also limit access and require the obstetrician to move away for a short time to allow the neonatal team access.

A specialized trolley which provides the facility for resuscitation with intact placental circulation at both caesarean births and assisted vaginal births may compromise the facility for both modes of birth, and separate designs may be preferable. Most assisted vaginal births do not take place in the caesarean room, so equipment customised for an assisted vaginal birth can remain in the labour ward delivery room, while equipment customised for caesarean births can remain in the operating room.

The cost of the equipment is obviously a serious consideration, but, given the enormous cost of the lifelong care of an individual with hypoxic birth injury [9], and the cost and effort of trying to mitigate any damage of hypoxic ischaemia at birth [25], even preventing a small proportion of injuries with resuscitation during an intact placental circulation could pay for the equipment many times over [9,25,26].

### 2.3. Heating and Prevention of Hypothermia

A standard resuscitation bed is equipped by an overhead radiant heater. The resuscitation platform of the trolley can also be warmed if the heater is switched on for a short time before the equipment is required, however this may be ineffective compared to radiant heat. The preterm neonate’s body is enclosed in polythene, a cap is placed on the neonatal head and warmed towels placed around its body. Chemical heating mattresses can be used to provide additional heating. Some types of mattress seem to be efficacious in maintaining normothermia (36.5–37.5 °C) even without an overhead radiant heater, but body temperature should be accurately checked in the delivery room. Instead, if such mattresses are used in combination with a radiant heater, hyperthermia (>38 °C) should be checked and avoided. The ambient temperature of the delivery room (23–25 °C, and >25° for newborns less than 28 wks) is important to reduce the risk of hypothermia even with all the above measures. The ambient temperature should be checked in the delivery room and promptly increased while assisting a very preterm delivery.

Providing the equipment for warming during these first few minutes after birth at motherside is a significant challenge. The possibility of the traditional radiant heater is limited at both caesarean section and vaginal births due to limited access immediately above the resuscitation platform. A temporary radiant heater could be used in positions 1 and 3, possibly attached to the operating light and only turned on immediately before the birth.

A warming mattress is available on one of the commercially available trollies, but, for preterm neonates, it may not be sufficient and supplementary chemical heating units have been effectively used. The ambient temperature of the air is extremely important and a warm theatre temperature is required for the safety of these neonates.

The use of a sterile polythene bag, covering also the head of the infant, presents no additional problem at caesarean section.

### 2.4. Equipment

Newborns who do not start breathing efficaciously after proper stimulation should be supported by nCPAP or positive pressure ventilation (PPV) within 1 min of life. In high-resource settings the initial provision of PPV is with a T-piece ventilator providing PEEP, (positive end expiratory pressure) with carefully controlled PIP (peak inspiratory pressure). A number of items of equipment are commercially available which provide PEEP and PIP with visible gauges showing the set pressure levels [27]. A suction device and warmed and humidified gas for the inspired gas is ideal. The user, or another member of the resuscitation team, controls the pressure levels during resuscitation. Resuscitation with PPV is normally started with air in term newborns and between 21% and 30% of oxygen in preterm newborns, then oxygen requirement is adjusted on newborn’s saturation values, so an increased concentration of oxygen needs to be available via a blender. Again, oxygen concentration is displayed and controlled by the clinician or another member of the resuscitation team if necessary.

The equipment to provide ventilation and suction can be remote or within the structure supporting the platform. International guidelines for neonatal resuscitation do not recommend routine oral, nasal, or oropharyngeal suctioning of the newborn at birth [20], and equipment could be useful only in case of suspected airway obstruction during positive pressure ventilation. Since some supporting framework is essential for all four positions, it seems logical, if possible, to include the ventilation equipment within the framework. If the equipment is remote then adjustment and monitoring of the pressure, flow and oxygen level by the neonatologist providing PPV is not feasible and requires a second person taking instructions. It also requires tubing from the remote equipment to the resuscitation platform which has an additional risk of compromising the sterile field. Providing all the equipment within the standard roomside resuscitation trolley into the motherside trolley is a considerable design challenge. Standard resuscitation equipment has centralised gas supply of air and oxygen and suction with an emergency backup of cylinders of air and oxygen. The gas supply for the ventilatory equipment is also a challenge. Tubing can be brought from standard outlets but these are cumbersome and limit the mobility of the trolley. They present a hazard within the theatre. Small cylinders can also be fitted but these are inevitably heavy and have a very limited supply.

### 2.5. Monitoring

Increasingly, more sophisticated monitoring is being used routinely. The minimal requirement is for heart rate and lung air entry via a stethoscope and also pulse oximetry. Electronic equipment for monitoring the neonatal parameters such as heart rate and oxygen saturation at birth are rapidly developing. In terms of size, weight, and sterility, none of these should present any difficulty in having them available on a mobile resuscitation trolley, but still they have not. Wireless transmission of signals makes it possible to include the equipment within the caesarean section sterile field if this can be contained within a sterile container.

### 2.6. Mobility

Roomside neonatal resuscitation trollies are generally stable and mobile enough so that if extended care is required the neonate can be moved to the special care nursery on the trolley. This is provided there is an independent gas supply on the machine. However, a transport incubator is frequently used to move the newborn from delivery room to neonatal intensive care unit.

Motherside neonatal resuscitation trolley is not intended to be used to move the neonate, but being mobile, it must also be capable of having a safe and stable platform for resuscitation of the neonate. Stability normally requires a large base but a large base limits mobility and proximity to the operating table.

## 3. Conclusions

Although the intervention of early cord clamping has no advantage for the mother or the baby, it is a reality that neonatal resuscitation was introduced while the practice was already established. Current practice and guidelines are based on the premise that there is unrestricted access to the neonate. The World Health Organisation (WHO) suggests that resuscitation with the cord intact is dependent upon the skill of the neonatologist [1]. A change in practice to remove the harmful intervention of early cord clamping in conjunction with neonatal resuscitation will require other changes in practice and the development of equipment that supports resuscitation procedures close to the mother and the obstetric team delivering the neonate. Two customised trollies are available commercially [28,29,30] but have not been obtained by most units throughout Europe and the USA.

## Figures and Tables

**Table 1:** Basic features to be assured in motherside neonatal resuscitation.

1. A stable flat, soft, but firm surface on which to place safely the neonate during resuscitation.
2. Warming equipment to prevent hypothermia. Preterm babies placed in a polythene bag while under the radiant heater are usually able to maintain their body temperature.
3. Good lighting
4. All the equipment to provide positive pressure ventilation with positive end-expiratory pressure (PEEP) immediately and easily available. The neonatologist can personally adjust the settings and oxygen levels if necessary. Suction is available. Other accessory equipment possibly available in store compartments.
5. Monitoring equipment available or accessible on or immediately near the trolley.
6. Easy access to the trolley allowing a team to provide resuscitation.

## Data Availability

No new data were created or analyzed in this study. Data sharing is not applicable to this article.
